# First-dollar cost-sharing for skilled nursing facility care in medicare advantage plans

**DOI:** 10.1186/s12913-017-2558-8

**Published:** 2017-08-29

**Authors:** Laura M. Keohane, Regina C. Grebla, Momotazur Rahman, Dana B. Mukamel, Yoojin Lee, Vincent Mor, Amal Trivedi

**Affiliations:** 1 Department of Health Policy, Vanderbilt University School of Medicine, 2525 West End Ave, Suite 1200, Nashville, TN 37203 USA; 2 Department of Health Services, Policy and Practice, Brown University, 121 South Main Street, Providence, RI 02903 USA; 3 Department of Medicine, Division of General Internal Medicine, University of California, Irvine, 100 Theory, Suite 120, Mail Code: 1835, Irvine, CA 92697 USA; 4 Center of Innovation in Long-Term Services and Supports for Vulnerable Veterans, Providence VA Medical Center, Providence, RI USA

**Keywords:** Medicare advantage, Post-acute care, Managed care, Cost-sharing, Skilled nursing facilities

## Abstract

**Background:**

The initial days of a Medicare-covered skilled nursing facility (SNF) stay may have no cost-sharing or daily copayments depending on beneficiaries’ enrollment in traditional Medicare or Medicare Advantage. Some policymakers have advocated imposing first-dollar cost-sharing to reduce post-acute expenditures. We examined the relationship between first-dollar cost-sharing for a SNF stay and use of inpatient and SNF services.

**Methods:**

We identified seven Medicare Advantage plans that introduced daily SNF copayments of $25-$150 in 2009 or 2010. Copays began on the first day of a SNF admission. We matched these plans to seven matched control plans that did not introduce first-dollar cost-sharing. In a difference-in-differences analysis, we compared changes in SNF and inpatient utilization for the 172,958 members of intervention and control plans.

**Results:**

In intervention plans the mean annual number of SNF days per 100 continuously enrolled inpatients decreased from 768.3 to 750.6 days when cost-sharing changes took effect. Control plans experienced a concurrent increase: 721.7 to 808.1 SNF days per 100 inpatients (adjusted difference-in-differences: −87.0 days [95% CI (−112.1,-61.9)]). In intervention plans, we observed no significant changes in the probability of any SNF service use or the number of inpatient days per hospitalized member relative to concurrent trends among control plans.

**Conclusions:**

Among several strategies Medicare Advantage plans can employ to moderate SNF use, first-dollar SNF cost-sharing may be one influential factor.

**Trial registration:**

Not applicable.

**Electronic supplementary material:**

The online version of this article (10.1186/s12913-017-2558-8) contains supplementary material, which is available to authorized users.

## Background

Medicare spending on post-acute services doubled between 2001 and 2013, and, according to an Institute of Medicine report, post-acute care accounts for nearly three-quarters of the unexplained geographic variation in Medicare expenditures [1, 2]. Under the traditional Medicare benefit, the first 20 days of post-acute care in a skilled nursing facility (SNF) are covered without cost-sharing, raising concerns about patients’ incentives to overuse these services even when they are of little value. Multiple proposals have recommended increasing Medicare’s copayments for post-acute care, which may reduce spending on potentially unnecessary services [3]. However, there is little evidence specific to post-acute care among older or disabled individuals to guide policymakers about this strategy’s potential impact.

Precedents for first-dollar SNF cost-sharing requirements can be found within the Medicare Advantage (MA) program. As of 2016, over 30% of Medicare beneficiaries opted to receive their health insurance coverage from a private MA plan rather than have traditional Medicare benefits [4]. Compared to the uniform cost-sharing requirements for traditional Medicare, MA plans have more discretion over post-acute cost-sharing levels, as well as the ability to require prior authorization and restrict provider networks. In contrast to traditional Medicare, MA plans may charge copayments starting on the first day of a SNF admission. Changes over time in MA plans’ SNF benefits are an opportunity to examine the relationship between cost-sharing and SNF utilization.

When evaluating first-dollar cost-sharing for SNF care in MA plans, three factors are important to consider. First, increasing SNF cost-sharing may adversely impact health outcomes and increase use of other health services. The RAND Health Insurance Experiment found that cost-sharing reduced individuals’ use of both necessary and unnecessary services. On the whole, these reductions in health care use generally did not harm health with the important exception of a sicker, low-income cohort. However, the study excluded older people and did not evaluate the use of post-acute services [5–7]. When Medicare beneficiaries faced greater copayments for outpatient care and prescription drugs, they reduced their use of these services but increased their use of expensive inpatient care [8, 9]. Similar unintended consequences could occur if reductions in SNF services due to cost-sharing are offset by longer hospital stays or more frequent rehospitalizations. Furthermore, compared to the services examined in the RAND study, it is not clear how much discretion beneficiaries have in using SNF services. Decisions about the need for post-acute care and length of SNF stay may be heavily influenced by providers’ recommendations. Second, because enrollment in a MA plan is voluntary, beneficiaries with serious health needs may avoid MA plans that require larger copayments for SNF care [10]. Plans with high cost-sharing for SNF care may realize lower rates of SNF use because beneficiaries who anticipate using these services opt for other MA plans or traditional Medicare. Without considering how SNF cost-sharing might influence beneficiaries’ plan enrollment choices, estimates of the impact of post-acute cost-sharing on utilization may be biased.

The third factor is the relationship between inpatient use and skilled nursing facility care. Even though MA plans can opt to cover SNF services without an inpatient stay, most beneficiaries will probably not require SNF services until after a hospitalization. This complementary relationship suggests that MA plans that can deter preventable hospitalizations among their members may also avoid unnecessary use of SNF services. On the other hand, SNF services can also potentially be a substitute for inpatient care in situations where inpatients may be able to transfer to a SNF rather than extend a hospital stay. For example, when traditional Medicare implemented prospective payments for hospital stays in the 1980s, the average inpatient length of stay declined while use of SNF services escalated [11–13].

Our study examines how SNF and inpatient utilization changed in MA plans that introduced daily copayments beginning on the first day of a SNF stay. We compare changes in utilization for these plans’ members to members of MA plans with no first-dollar cost-sharing for a SNF stay. Beneficiaries may respond to first-dollar SNF cost-sharing by reducing their length of stay since out-of-pocket costs accumulate immediately. Alternatively, some beneficiaries may forego a SNF admission entirely. To analyze whether our results are influenced by selective disenrollment, we consider outcomes among members who remained in their MA plan as well as those who disenrolled when cost-sharing changes took effect. Our results provide new evidence about the potential impact of first-dollar cost-sharing on post-acute nursing home utilization and MA enrollment choices.

## Methods

### Data sources

The Medicare Healthcare Effectiveness Data and Information Set (HEDIS), linked to the Medicare Beneficiary Summary File, provided individual-level data on MA plan enrollment, MA inpatient use, and demographic characteristics for the years 2007-2010. To measure SNF utilization, we linked beneficiaries to a Residential History File built from Minimum Data Set (MDS) assessments for all individuals admitted to a Medicare-certified nursing home, including MA enrollees [14]. The MDS includes use of SNF services among both traditional Medicare and MA enrollees. Traditional Medicare claims captured inpatient use for beneficiaries who left the MA program.

### Study population

Based on MA plans’ annual SNF benefit descriptions in the Centers for Medicare and Medicaid Services (CMS) Plan Finder Data, we identified intervention plans that introduced first-dollar cost-sharing, which we defined as daily copayments for at least the first five days of a SNF stay. The year before and after copayment changes are identified as the “baseline” and “post” year, respectively. We matched intervention plans to control plans that required no first-dollar cost-sharing for SNF care in the baseline and post year. Intervention and control plans in a matched pair had the same tax status and were in the same state or, if a control could not be identified in the same state, the same Census region.

We identified 20 MA plans with HEDIS data that introduced first-dollar SNF cost-sharing requirements between 2007 and 2010. To meet study inclusion criteria, intervention and control plans had to have at least 50 inpatients with SNF use in either the baseline or post year. We excluded Special Needs Plans and plans that required coinsurance for SNF services or changed whether a hospital stay was required prior to a SNF stay. After applying these exclusion criteria and excluding one intervention plan with no suitable control plan, our final sample included 7 intervention plans with matched controls. Final intervention plans added copayments in 2009 or 2010. All intervention plans had control plans that operated in the same state or in neighboring states, except for 1 plan that was matched to a control plan that operated in the same Census region.

The common factor across all intervention and control plans was whether they required first-dollar SNF cost-sharing or not. Other aspects of benefits could vary within and across plans. In intervention plans, cost-sharing requirements for a week of inpatient care increased for most members (96% of enrollees) in the same year that SNF cost-sharing increased. Matched control plans did not make changes to their inpatient cost-sharing.

We disregarded changes in SNF cost-sharing requirements for days 6 and beyond of a SNF stay. Prior to introducing first-dollar cost-sharing, intervention plans varied in how many days of a SNF stay were not subject to copayments (range: the first 5 to 20 days). During the baseline and post year, three control plans had no copayments for the first 20 days of a SNF stay; another had no copayments for the first 10 days. Given this variation in cost-sharing requirements for days 6-20, we could not analyze how this aspect of SNF cost-sharing could influence results. We focus on examining the relationship between first-dollar SNF cost-sharing and health care use, which is averaged across plans’ different benefit designs.

Our main analysis includes intervention and control plan members continuously enrolled in the baseline and post year, as well as decedents in either year. We excluded beneficiaries who started the year with full Medicaid coverage. Although we present results for all continuously enrolled members, we focus on results for inpatients as our primary set of outcomes because SNF care usually follows a hospital stay. To examine the relationship between SNF cost-sharing and intensity of use of SNF services, we also present results for average SNF length-of-stay among inpatients with any SNF use.

We also consider the results for inpatients to be a more conservative estimate of the relationship between first-dollar SNF cost-sharing and SNF utilization. If increased inpatient cost-sharing in intervention plans reduced hospitalization rates among all members in the post year, then SNF use may have declined among all members regardless of whether there were any changes to SNF benefits, which would overstate the effect of SNF cost-sharing. In contrast, the difference-in-differences estimate among inpatients could be attenuated for two reasons. First, increased inpatient cost-sharing may have decreased hospitalizations among healthier patients whose need for inpatient care was marginal. Compared to the pre-year period, inpatients in the post-period might be sicker on average and more in need of post-acute services. Second, higher inpatient cost-sharing requirements in the post year might encourage inpatients to have a shorter length of stay in the hospital and transfer to a SNF. Conditional on having a hospital stay, the increases in inpatient cost-sharing might be expected to increase SNF use among inpatients, which would offset any decreases in SNF use due to first-dollar SNF cost-sharing. In that context, our difference-in-differences estimate among inpatients will be biased towards the null and more conservative than the estimate among all members.

If beneficiaries left intervention plans to avoid increased SNF cost-sharing, then our results could be biased by analyzing results for continuously enrolled members. To investigate whether selective disenrollment influenced our findings, we performed a sensitivity analysis that included beneficiaries who were enrolled in an intervention or control plan for the baseline year but not for the entire post year. These disenrollees switched to other MA plans (including plans that are not intervention or control plans) or the traditional Medicare program. We measured inpatient and SNF service use for members after they left intervention and control plans. If a plan was no longer offered in a member’s residential area during the post year, we excluded those disenrollees (*n* = 227). That exclusion criterion was determined by identifying counties where more than 90% of plan members left.

### Outcomes

Our two binary outcomes included any use of inpatient or SNF services. Count outcomes included the annual number of inpatient days, the annual number of SNF days, and, conditional upon having any SNF use, SNF length of stay. If a beneficiary had more than one SNF stay per year, this latter variable averaged length of stay across all the beneficiary’s SNF stays. When we measured the number of SNF days per beneficiary and SNF length of stay, we capped the length of stay for each SNF admission at 100 days to be consistent with the duration of traditional Medicare coverage for a SNF episode. For SNF stays that extended from December into January, we capped the length of stay at the end of December to accurately reflect the time period when copayments were in effect. We disregarded any transfers between nursing homes in estimating length of stay.

### Analysis

A difference-in-differences analysis compared intervention and control plan members’ changes in utilization at the individual level. We used a generalized linear model with a binomial link for binary outcomes and a negative binomial link for count outcomes. We fit our models using generalized estimating equations (GEE) with exchangeable correlation to account for repeated measurements among individuals in the same plan for multiple years. An alternative modeling approach that clustered standard errors by hospital referral region did not substantially change our results.

The model includes an indicator variable for intervention plans, an indicator variable for the post year and an interaction term between these two variables. Our adjusted difference-in-differences estimate is the average marginal effect of this interaction term [15]. Covariates included beneficiaries’ age, sex, race, use of the Part D subsidy or limited Medicaid, and a fixed effect for each intervention-control pair. To account for decedents with partial year enrollment, our model included a continuous variable for the number of months alive.

As a sensitivity analysis, we estimated separate results for each pair and for several subpopulations (multiple age groups, males, females, white beneficiaries, black beneficiaries and those with financial assistance for Medicare costs).

Analyses were conducted using SAS 9.4 and Stata 14. The study protocol was approved by Brown University’s Human Research Protections Office, Vanderbilt University’s Institutional Review Board, and the CMS Privacy Board.

## Results

Intervention MA plans initiated first-dollar SNF copayments ranging from $25 to $150 per day. Cost-sharing for a week of inpatient care also increased by an average of $69 per day (range: $0 to $157) across all intervention plan members. As of the baseline year, our main study population – continuously enrolled members and decedents— included 75,044 intervention plan members and 97,914 control plan members. Members of plans that introduced copayments were more likely to be black and more likely to receive limited Medicaid or Part D subsidies (Table 1).

**Table 1:** Baseline characteristics of medicare advantage members enrolled in plans that did and did not add first-dollar cost-sharing for skilled nursing facility care

	Intervention plans	Control plans
Number of beneficiaries	75,044	97,914
Age (%): Age < 65	10.2	7.4***
Age 65-74	45.4	43.9
Age 75-84	33.0	37.5
Age 85 and over	11.3	11.1
Female (%)	57.5	56.6**
Race (%): White	76.0	89.9***
Black	19.3	5.4
Other	4.7	4.7
Financial assistance (%): None	86.6	91.0***
Part D Low Income Subsidy	6.5	4.5
Limited Medicaid	7.0	4.4
Mean daily copayments (range), in U.S. Dollars:		
SNF, days 1–5	0.0 (0–0)	0.0 (0–0)
SNF, days 6–20	28.2 (0–93)	14.7 (0–87)***
Inpatient, days 1–7	32.5 (0–214)	88.1 (0–205)***

Based on authors’ analysis of data from Medicare Advantage and Medicare enrollment records. The study included seven intervention plans, which added first-dollar SNF cost-sharing, and seven matched control plans, which maintained having no first-dollar SNF cost-sharing for the entire study period. Percentages may not sum to 100 due to rounding. For each intervention plan and its matched control, the baseline year was the year before the intervention plan added first-dollar cost-sharing. SNF = Skilled nursing facility. We tested for significant differences between intervention and control plans using chi-square tests for categorical variables and t-tests for continuous variables. **p< 0.01 ***p < 0.001

In intervention plans, the proportion of continuously enrolled members who were hospitalized declined from 18.8% in the year before the cost-sharing was introduced to 17.8% in the year after cost-sharing (Table 2). However, inpatient use increased in control plans by 1.1 percentage points, indicating that intervention plans had a significant reduction in inpatient use relative to control plans (adjusted difference-in-differences −1.6 percentage points [95% CI (−2.0,-1.1)]. The percentage of continuously enrolled members with any SNF use stayed steady at 4.0% in intervention plans, compared to an increase from 4.1 to 4.8% in control plans (adjusted difference-in-differences −0.4 percentage points [95% CI (−0.7,-0.2)]). Intervention plans also had relative reductions in the mean annual number of inpatient days and SNF days per 100 members (adjusted difference-in-difference estimates: −12.1 days [95% CI (−14.5, −9.7)] and −26.7 days [95% CI (−29.7, −23.8)], respectively). These results changed in two ways when we limited our study population to continuously enrolled members with inpatient use. First, conditional on having an inpatient stay, intervention and control plans no longer had significantly different growth in mean annual inpatient days (adjusted difference-in-differences: 8.4 days per 100 inpatients [95% CI -18.0, 34.8]). Second, introducing SNF copayments was no longer significantly associated with a reduction in the probability of using any SNF care. However, intervention plans still experienced a relative reduction in the mean number of SNF days among inpatients. In intervention plans the number of SNF days per 100 inpatients declined from an average of 768.3 days to 750.6 days when cost-sharing changes took effect. In contrast, SNF use increased in control plans from 721.7 to 808.1 SNF days per 100 inpatients (adjusted difference-in-differences: -87.0 [95% CI -112.1,-61.9]). Detailed results from the regression models for all members and inpatients are presented in Additional file 1: Tables A1 and A2.

**Table 2:** Change in inpatient and skilled nursing facility use among continuously enrolled members in intervention plans versus those in control plans

	Intervention plans		Control plans		Difference-in-differences	
	Year prior to copayment increases	Change	Year prior to copayment increases	Change	Unadjusted	Adjusted (95% CI)
All Members						
Any inpatient use (%)	18.8	−1.0	15.4	1.1	−2.0	−1.6*** (−2.0, −1.2)
Any SNF use (%)	4.0	0.0	4.1	0.6	−0.6	−0.4*** (−0.7,-0.2)
Annual inpatient days per 100 enrollees	178.6	−11.1	105.0	8.1	−19.1	−12.1*** (−14.5,-9.7)
Annual SNF days per 100 enrollees	157.3	−8.0	132.2	26.1	−34.2	−26.7*** (−29.7,-23.8)
Inpatients						
Any SNF use (%)	19.5	0.8	23.0	2.1	−1.3	−0.9 (−2.2, 0.3)
Annual inpatient days per 100 hospitalized enrollees	949.9	−10.4	681.9	4.4	−14.9	8.4 (−18.0, 34.8)
Annual SNF days per 100 hospitalized enrollees	768.3	−17.7	721.7	86.4	−104.1	−87.0***(−112.1,-61.9)

Authors’ analysis of data on hospital utilization from the Healthcare Effectiveness Data and Information Set and data on skilled nursing facility utilization from the Residential History File from the Minimum Data Set. SNF = Skilled nursing facility. ***p < 0.001

Among inpatients with SNF use, intervention plans also experienced significantly larger decreases in mean SNF length of stay than control plans. This measure declined from 32.6 days to 30.2 days when intervention plans introduced first-dollar cost-sharing changes, but only declined by 0.3 days in control plans (adjusted difference-in-differences: -2.2 [95% CI: -4.2, −0.3], Additional file 1: Table A3).

**Fig. 1:**
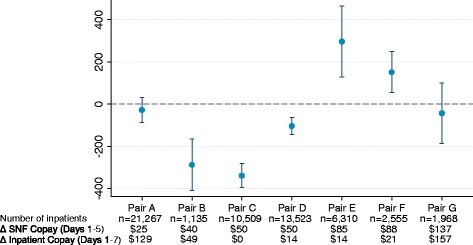
Adjusted Difference-in-Differences Estimates in Number of Skilled Nursing Facility Days per Year among Inpatients in Intervention Plans versus Those in Control Plans, by Matched Pair. Notes: Authors’ analysis of data on hospital utilization from the Healthcare Effectiveness Data and Information Set and data on skilled nursing facility utilization from the Residential History File from the Minimum Data Set. The point estimates and 95% confidence intervals (represented by the whiskers) refer to the adjusted difference-in-differences for number of skilled nursing facility days per year among enrollees in each intervention-control pair in the study. Estimates were adjusted for age, sex, race, and receipt of Medicaid or low-income subsidy. SNF = Skilled nursing facility

To explore the changes in SNF days among inpatients in more detail, Fig. 1 presents the adjusted difference-in-differences estimates for this measure separately for each matched intervention-control pair. Results are mixed: three plans that introduced first-dollar cost-sharing had significant decreases in the mean number of SNF days per inpatient relative to their matched control plans. Another two pairs had negative, but insignificant, difference-in-differences estimates. The remaining two plans that introduced first-dollar cost-sharing had significant increases in SNF use relatively to their matched control plans. There are no indications that plans that introduced larger SNF copayments had greater reductions in the number of SNF days. There is also no clear pattern between the size of the increase in inpatient cost-sharing and the results for SNF utilization among inpatients. Results were fairly consistent when we performed stratified analyses by several population characteristics: age, sex, race, receipt of financial assistance (Additional file 1: Figure S1).

In the year following the introduction of copayments, 26% of the members of intervention plans exited the plan. In five out of seven intervention plans, over 25% of members enrolled during the baseline year and still alive as of the end of the baseline year were not enrolled for the entire post year. In contrast, only 8% of control plan members left their plan after the baseline year (*p* < 0.001, Additional file 1: Table A4). Members who disenrolled had higher rates of SNF use in the baseline year compared to members who stayed in their plans: 3.3% of disenrollees had prior SNF use compared to 2.9% of continuously enrolled members in intervention plans (*p* < 0.01); the corresponding comparison in control plans was 4.9% versus 3.3% (*p* < 0.001).

**Fig. 2:**
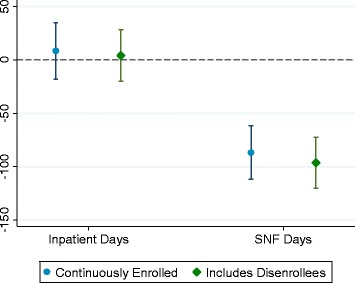
Adjusted Difference-in-Differences Estimates in Number of Skilled Nursing Facility Days per Year among Inpatients in Intervention Plans versus Those in Control Plans, by Members’ Disenrollment Status after Cost-sharing Changes. Notes: Authors’ analysis of data on hospital utilization from the Healthcare Effectiveness Data and Information Set and Medicare claims; data on skilled nursing facility utilization from the Residential History File from the Minimum Data Set. The point estimates and 95% confidence intervals (represented by the whiskers) refer to the adjusted difference-in-differences for number of skilled nursing facility days per year among enrollees in each intervention-control pair in the study. Estimates were adjusted for age, sex, race, and receipt of limited Medicaid or Part D low-income subsidy. SNF = Skilled nursing facility

To assess whether first-dollar SNF cost-sharing reduced SNF use by prompting potential users of SNF services to leave intervention plans, we repeated our analyses after including disenrollees. If disenrollees from intervention plans had disproportionately high rates of SNF utilization in the post year, then the difference-in-differences estimate could be attenuated. Despite the large membership turnover in intervention plans, the results were unchanged (Fig. 2, adjusted difference-in-difference estimate for mean SNF days per 100 inpatients: −96.3 days [95% CI -120.1, −72.4]).

## Discussion

In MA plans that introduced first-dollar cost-sharing for SNF stays, we observed corresponding decreases in the duration of SNF use among hospitalized members. Daily copayments of $25 to $150 were associated with an absolute reduction of 87 SNF days per 100 inpatients or a relative reduction of 11% compared to intervention plans’ average SNF utilization in the baseline year (768.3 SNF days per 100 inpatients). Moreover, we found no offsetting increases in hospital use following the introduction of SNF copayments.

Plans that introduced first-dollar SNF cost-sharing had disenrollment rates that were three times greater than control plans. In both intervention and control plans, beneficiaries who disenrolled were more likely to have SNF use in the baseline year compared to continuously enrolled beneficiaries. However, our results were unchanged when our analysis included disenrollees and their health care use throughout the study period. This finding suggests that intervention plans’ reduction in SNF use is not attributable to selective disenrollment of potential SNF users.

To the best of our knowledge, this study is the first examination of individuals’ response to first-dollar cost-sharing for SNF care. Despite extensive evidence that patients reduce health services use when exposed to greater cost-sharing, there is little data about price sensitivity for post-acute services. Traditional Medicare beneficiaries are more likely to leave SNFs when copayments begin on the 21st day and when benefits are exhausted on the 100th day [16]. Our findings focus on cost-sharing for the start of a SNF stay, when beneficiaries may have few alternatives to SNF services and may be less responsive to cost-sharing. Whether reductions in SNF use impact health outcomes is still unknown, partly because there is little evidence regarding post-acute care treatment guidelines [17]. When traditional Medicare switched to a prospective per diem payment for SNF services, the likelihood of being admitted to a SNF dropped slightly, but there was no impact on length of stay [18–20]. There is little data on how these shifts impacted beneficiaries’ health.

One significant challenge in interpreting our results is the fact that most beneficiaries faced simultaneous increases in inpatient cost-sharing at the same time first-dollar SNF cost-sharing was introduced. Among all plan members, we observed relative declines in whether members of intervention plans had any inpatient use and in the number of inpatient days per member. These inpatient trends may partly explain why intervention plans also had less growth in SNF use among all members relative to control plans. However, by focusing on the results for inpatients as our main set of study outcomes, we attempt to mitigate the concern that we are overstating the impact of SNF cost-sharing increases. Once a beneficiary is already hospitalized, an increase in inpatient cost-sharing may be more likely to increase the use of SNF services as a substitute for another day in the hospital. The impact of introducing first-dollar SNF cost-sharing may differ in a situation where there are no changes to inpatient cost-sharing.

Several proposals for reducing post-acute spending have advocated charging traditional Medicare beneficiaries a copayment upon entry to a SNF [21]. Our results may not generalize to traditional Medicare, which lacks MA plans’ ability to require prior authorization and impose network restrictions. MA members have historically been healthier than traditional Medicare beneficiaries and, on average, may have different price sensitivity to SNF copay changes.

Our results also contribute to debates about selective disenrollment from the MA program, where members with greater use of health care services and post-acute care use are more likely to switch to traditional Medicare than MA members who use fewer services [22, 23]. In 2012, 10 % of MA members voluntarily switched to another MA plan or traditional Medicare [24]. We found comparable frequency of disenrollment in control plans (8%). However, we observed greater disenrollment in intervention plans (26%) and disenrollees were disproportionately low-income Medicare beneficiaries with partial Medicaid. Several unmeasured factors may have contributed to beneficiaries’ enrollment decisions, including new MA plans entering the market or other changes in benefits. New regulations, effective in 2011 after our study’s time period, began limiting out-of-pocket costs for key services, including SNF care, to prevent MA plans from using benefit design as a means for discouraging enrollment of high-cost patients [25, 26]. The caps on SNF cost-sharing have become more restrictive over time, and, as of 2015, MA plans cannot require first-dollar cost-sharing for SNF services unless they meet certain guidelines for limiting beneficiaries’ out-of-pocket costs across all medical services [27].

Given the observational nature of our study, there are several important limitations to note about how beneficiaries’ SNF use may have been influenced by factors other than the introduction of SNF copayments. MA plans could have adopted other strategies, unobservable in our data, to manage SNF use at the same time they introduced first-dollar cost-sharing, such as increased use of prior authorization requirements for SNF services or care management strategies to reduce SNF length of stay. If plans that implemented first-dollar cost-sharing were more concerned about reducing SNF utilization, they may have been more likely to implement such strategies. We do not examine whether intervention and control plans had similar long-term trends in SNF use prior to the introduction of copayments because we have limited data on some plans prior to the baseline year and one plan made copayment changes prior to the baseline year. We were able to match intervention and control plans on the basis of geographic location and tax status, but limited the number of factors to include in the matching strategy to include a broader sample of intervention and control plans. The sample of seven plans limited analyses examining responsiveness by amount of copayment. Because we do not have MA claims to verify plans’ payments for SNF care, members may have paid out-of-pocket for days in a nursing home that were not covered by MA plans, such as days where custodial care was provided instead of SNF services. Finally, the populations in our intervention and control plans showed significant differences in demographic characteristics, which may reflect underlying differences in health characteristics that we cannot observe in our data sources. However, we controlled for observable differences in characteristics and our results were consistent across several subpopulations.

## Conclusions

Our findings suggest that introducing SNF copayments in MA plans may moderate use of SNF services, but we cannot draw any definitive conclusions about whether SNF cost-sharing was the only factor that influenced the use of SNF services among our study population. Further study is needed to disentangle the roles of SNF and inpatient cost-sharing, as well as care management approaches for post-acute care. We found no indications that inpatient use increased when first-dollar SNF cost-sharing was introduced, which suggests cost-sharing for this service may have reduced spending in these plans. Cost-sharing changes also may have prompted enrollees to exit their plan, but even after accounting for the possibility of selective disenrollment among likely SNF users, we still found an association between first-dollar cost-sharing and reducing use of SNF care. Benefit design for post-acute services may have a significant impact on enrollment choices in MA plans and utilization of services.

## Additional file

Additional file 1: This file contains the appendix figures and tables referenced in the manuscript. (DOC 101 kb)

## Abbreviations

CMS: Centers for medicare and medicaid services
GEE: Generalized estimating equations
HEDIS: Healthcare effectiveness data and information set
MA: Medicare advantage
MDS: Minimum data set
SNF: Skilled nursing facilities
