# Supplementary material for: First-dollar cost-sharing for skilled nursing facility care in medicare advantage plans
Source: BMC Health Serv Res. 2017 Aug 29;17:611. doi: 10.1186/s12913-017-2558-8 (PMC5576284; doi:10.1186/s12913-017-2558-8)
Supplement: Additional file 1: — This file contains the appendix figures and tables referenced in the manuscript. (DOC 101 kb) [file 12913_2017_2558_MOESM1_ESM.doc]

Appendix Table A1: Model Results for All Members

|  | Any inpatient use | | Any SNF use | | Number of inpatient days | | Number of SNF days | |
| --- | --- | --- | --- | --- | --- | --- | --- | --- |
|  | Coeff. | Std. Err. | Coeff. | Std. Err. | Coeff. | Std. Err. | Coeff. | Std. Err. |
| Intervention plan | 0.17*** | (0.01) | -0.09*** | (0.03) | 0.30*** | (0.01) | -0.08*** | (0.01) |
| Post Year | 0.07*** | (0.01) | 0.11*** | (0.02) | 0.09*** | (0.01) | 0.16*** | (0.01) |
| Intervention plan * Post Year | -0.12*** | (0.02) | -0.11** | (0.03) | -0.08*** | (0.01) | -0.16*** | (0.01) |
| Female | -0.12*** | (0.01) | 0.12*** | (0.02) | -0.17*** | (0.01) | 0.12*** | (0.01) |
| Age | 0.04*** | (0.00) | 0.08*** | (0.00) | 0.03*** | (0.00) | 0.10*** | (0.00) |
| Under Age 65 * Age | 0.01*** | (0.00) | 0.01*** | (0.00) | 0.01*** | (0.00) | 0.02*** | (0.00) |
| White |  |  |  |  |  |  |  |  |
| Black | 0.00 | (0.02) | -0.02 | (0.03) | 0.16*** | (0.01) | 0.18*** | (0.01) |
| Other | -0.23*** | (0.03) | -0.47*** | (0.05) | -0.19*** | (0.01) | -0.43*** | (0.01) |
| Part D Limited Income Subsidy | 0.28*** | (0.02) | 0.29*** | (0.04) | 0.29*** | (0.01) | 0.49*** | (0.01) |
| Partial Medicaid | 0.34*** | (0.02) | 0.35*** | (0.04) | 0.34*** | (0.01) | 0.62*** | (0.01) |
| Months alive per year | -0.24*** | (0.00) | -0.17*** | (0.00) | -0.26*** | (0.00) | -0.22*** | (0.00) |
| Constant | -1.46*** | (0.07) | -7.62*** | (0.11) | 0.90*** | (0.03) | -5.00*** | (0.04) |

Notes: Table presents coefficients from a generalized linear model with a binomial link for binary outcomes and a generalized linear model with a negative binomial link for count outcomes. Models also included fixed effects for each matched pair. Authors’ analysis of data on hospital utilization from the Healthcare Effectiveness Data and Information Set and data on skilled nursing facility utilization from the Residential History File from the Minimum Data Set. SNF=Skilled nursing facility. *p < 0.05 **p < 0.01 ***p < 0.001.

Appendix Table A2: Model Results for Inpatients

|  | Any SNF use | | Number of SNF days | |
| --- | --- | --- | --- | --- |
|  | Coeff. | Std. Err. | Coeff. | Std. Err. |
| Intervention plan | -0.17*** | (0.03) | -0.11*** | (0.01) |
| Post Year | 0.10*** | (0.03) | 0.12*** | (0.01) |
| Intervention plan * Post Year | -0.06 | (0.04) | -0.12*** | (0.02) |
| Female | 0.28*** | (0.02) | 0.19*** | (0.01) |
| Age | 0.06*** | (0.00) | 0.06*** | (0.00) |
| Under Age 65 * Age | 0.01*** | (0.00) | 0.01*** | (0.00) |
| White |  |  |  |  |
| Black | -0.02 | (0.04) | 0.20*** | (0.01) |
| Other | -0.38*** | (0.06) | -0.24*** | (0.02) |
| Part D Limited Income Subsidy | 0.16*** | (0.04) | 0.29*** | (0.02) |
| Partial Medicaid | 0.18*** | (0.04) | 0.39*** | (0.02) |
| Months alive per year | -0.03*** | (0.00) | -0.02*** | (0.00) |
| Constant | -5.65*** | (0.13) | -2.93*** | (0.06) |

Notes: Table presents coefficients from a generalized linear model with a binomial link for binary outcomes and a generalized linear model with a negative binomial link for count outcomes. Models also included fixed effects for each matched pair. Authors’ analysis of data on hospital utilization from the Healthcare Effectiveness Data and Information Set and data on skilled nursing facility utilization from the Residential History File from the Minimum Data Set. SNF=Skilled nursing facility. *p < 0.05 **p < 0.01 ***p < 0.001.

Appendix Table A3: Change in SNF Length of Stay among Continuously Enrolled Inpatients with SNF Use in Intervention Plans Versus Those in Control Plans

|  | Intervention Plans | | Control Plans | | Difference-in-differences | |
| --- | --- | --- | --- | --- | --- | --- |
|  | Year Prior to Copayment Increases | Change | Year Prior to Copayment Increases | Change | Unadjusted | Adjusted (95% CI) |
| Mean SNF length-of-stay in days | 32.6 | -2.5 | 26.6 | -0.3 | -2.2 | -2.2* ( -4.2, -0.3) |

Notes: Authors’ analysis of data on hospital utilization from the Healthcare Effectiveness Data and Information Set and data on skilled nursing facility utilization from the Residential History File from the Minimum Data Set. SNF=Skilled nursing facility. *p < 0.05 **p < 0.01 ***p < 0.001.

Appendix Figure 1. Adjusted Difference-in-Differences Estimates in Number of Skilled Nursing Facility Days per Year among Inpatients in Intervention Plans versus Those in Control Plans, by Selected Characteristics

Notes: Authors’ analysis of data on hospital utilization from the Healthcare Effectiveness Data and Information Set and data on skilled nursing facility utilization from the Residential History File from the Minimum Data Set. The point estimates and 95 percent confidence intervals (represented by the whiskers) refer to the adjusted difference-in-differences for number of skilled nursing facility days per year among enrollees in each intervention-control pair in the study. Estimates were adjusted for age, sex, race, and receipt of limited Medicaid or Part D low-income subsidy. SNF=Skilled nursing facility.

Appendix Table A4: Characteristics of Medicare Advantage members by Disenrollment Status
After Skilled Nursing Facility Cost-sharing Changes

|  | Intervention plans | | Control plans | |
| --- | --- | --- | --- | --- |
|  | Stayed Enrolled | Dis-enrolled | Stayed Enrolled | Dis- enrolled |
| Number of beneficiaries | 71,222 | 24,422 | 94,351 | 8,058 |
| Percent of beneficiaries | 74.5 | 25.5 | 92.1 | 7.9 |
| Age range (%): Age <65 | 10.4 | 14.7*** | 7.5 | 13.5*** |
| Age 65-74 | 46.6 | 45.5 | 44.8 | 47.2 |
| Age 75-84 | 32.7 | 30.8 | 37.3 | 29.5 |
| Age 85 and over | 10.3 | 9.0 | 10.3 | 9.8 |
| Female (%) | 57.8 | 60.6*** | 56.8 | 57.2 |
| Race (%): White | 76.0 | 73.2*** | 89.9 | 82.1*** |
| Black | 19.3 | 21.9 | 5.4 | 9.9 |
| Other | 4.7 | 4.9 | 4.7 | 8.1 |
| Financial assistance (%):   None | 86.9 | 77.5*** | 91.2 | 86.6*** |
| Part D Low Income Subsidy | 6.4 | 6.1 | 4.4 | 6.3 |
| Limited Medicaid | 6.7 | 16.5 | 4.3 | 7.2 |
| Mean daily copayments (range): |  |  |  |  |
| SNF, days 1-5 | 0 | 0 | 0 | 0 |
| SNF, days 6-20 | 28.1 | 31.8*** | 14.6 | 36.7*** |
| Inpatient, days 1-7 | 32.4 | 46.2*** | 88.3 | 88.4 |
| Health care use in year before copay changes: |  |  |  |  |
| Any hospitalization (%) | 16.1 | 16.7* | 13.6 | 14.2 |
| Mean total number inpatient days | 1.2 | 1.3* | 0.8 | 0.9 |
| Any SNF stay (%) | 2.9 | 3.3** | 3.3 | 4.9*** |
| Mean total number SNF days | 1.1 | 1.3* | 1.0 | 2.0*** |

Notes: Based on authors’ analysis of data from Medicare Advantage and Medicare enrollment records. Beneficiaries who died during the pre-year of the study are excluded. SNF=Skilled nursing facility. We tested for significant differences between intervention and control plans using chi-square tests for categorical variables and t-tests for continuous variables. *p < 0.05 **p < 0.01 ***p < 0.001.
